# Endovascular Treatment of Posterior Circulation Saccular Aneurysms With the p64 Flow Modulation Device: Mid-and Long-Term Results in 54 Aneurysms From a Single Center

**DOI:** 10.3389/fneur.2021.711863

**Published:** 2021-07-16

**Authors:** Victoria Hellstern, Marta Aguilar-Pérez, Elina Henkes, Carmen Serna-Candel, Christina Wendl, Hansjörg Bäzner, Oliver Ganslandt, Hans Henkes

**Affiliations:** ^1^Neuroradiologische Klinik, Kopf- und Neurozentrum, Klinikum Stuttgart, Stuttgart, Germany; ^2^Institut für Röntgendiagnostik, Zentrum für Neuroradiologie, Universitätsklinikum Regensburg, Regensburg, Germany; ^3^Neurologische Klinik, Neurozentrum, Klinikum Stuttgart, Stuttgart, Germany; ^4^Neurochirurgische Klinik, Neurozentrum, Klinikum Stuttgart, Stuttgart, Germany; ^5^Medizinische Fakultät der Universität Duisburg-Essen, Essen, Germany

**Keywords:** p64, posterior circulation aneurysms, flow diversion, unruptured aneurysm, saccular aneurysm

## Abstract

**Objective:** Flow diverter (FD) stents have become one of the most common tools for treating intracranial aneurysms; however, their role in treating posterior circulation aneurysms is still discussed with controversy. In this study, we evaluated the safety and effectiveness of p64 FD for the treatment of saccular, unruptured aneurysms in the posterior circulation over a long-term follow-up period in a single center.

**Methods:** From our prospectively maintained database, we retrospectively identified patients who underwent treatment of an intracranial saccular aneurysm arising from the posterior circulation with ≥1 p64 FD implanted or attempted between October 2012 and December 2019. Aneurysms could have been treated with prior or concomitant saccular treatment (e.g., coiling, intra-aneurysmal flow diversion). Aneurysms with parent vessel implants other than p64, fusiform aneurysms, and dissections were excluded. Peri- and postprocedural complications, clinical outcome, and clinical and angiographic follow-up results were evaluated.

**Results:** In total, 54 patients (45 female, 9 male; mean age 55.1 years) with 54 intracranial aneurysms met the inclusion criteria. In 51 cases (94.4%), one p64 was implanted; in 2 cases (3.7 %), two p64s were implanted; in one case, deployment of the p64 was not feasible. Procedural complications occurred in 3.7% and postprocedural complications in 9.3 %, respectively. Hemorrhagic complications occurred in 2/54 patients (3.7%), thereof one fatal parenchymal hemorrhage. Ischemic complications were observed in 5/54 patients (9.3%). Early, mid-term, and long-term angiographic follow-up examinations showed complete or near-complete aneurysm occlusion, defined according to the O'Kelly –Marotta (OKM) scale as OKM C + D in 56, 75.6, and 82.9 %, respectively. Asymptomatic side vessel occlusions occurred in 3.8%, each during the first follow-up.

**Conclusions:** The implantation of a p64 FD is a safe and effective device for endovascular treatment of posterior circulation saccular aneurysms with a high success rate and low morbi-mortality.

## Introduction

Flow diverter (FD) implantation has become one of the most common tools for treating intracranial aneurysms (IAs). In the last years, the number of available FDs has increased continuously. One of these FDs is the p64 FD (phenox, Bochum, Germany). Its unique feature is that it can be deployed entirely and resheated due to its mechanical detachment system allowing for potentially necessary repositioning. Several published studies have reported the long-term efficacy of the p64 (1–3). However, these studies have focused mainly or exclusively on aneurysms in the anterior circulation. There is only limited data for treating aneurysms with the p64 in the posterior circulation.

Furthermore, FD in the posterior circulation is still discussed more controversially due to higher rates of ischemic complications and morbidity and mortality (4–6).

In the present study, we report the experience of treating intracranial saccular unruptured aneurysms arising from the posterior circulation endovascularly with the p64 FD. To the best of our knowledge, this is the most extensive study to report data for saccular aneurysms in the posterior circulation treated exclusively with the p64 FD.

## Methods

### Device Description

The p64 Flow Modulation Device is braided from 64 nitinol wires and two platinum wires wrapped around opposing nitinol wires for visibility under x-ray fluoroscopy. At the proximal end, the 64 wires form 8 bundles, each carrying a radiopaque marker. The unique feature of the p64 is that after full deployment, it is entirely resheatable and repositionable due to its mechanical detachment. It is delivered via a 0.027-inch inner diameter microcatheter.

### Patient Population

We retrospectively reviewed our prospectively maintained database to identify all patients with intracranial saccular unruptured aneurysms in the posterior circulation treated with at least one p64 FD between October 2012 and December 2019. Inclusion criteria for this series were all aneurysms treated endovascularly with p64 implantation only or with p64 and simultaneous or prior coiling. Only patients with unruptured aneurysms or past the acute stage of SAH (>30 days) were included. Fusiform, dissecting, or blister-like aneurysms, as well as dolicho-ectatic basilar artery aneurysms, were excluded. Aneurysms treated with other parent vessel implants such as other FDs or stents were also excluded. We recorded demographic data, anatomical features, location of the aneurysm, procedural and post-procedural complications, and clinical and angiographic outcomes according to the latest available follow-up for each of these patients.

### Endovascular Procedure

In each case, patients had given written informed consent at least 24 hours before the procedure after being informed about the intended treatment and potential alternatives.

All treatments were performed under general anesthesia using bi-plane digital subtraction angiography (DSA) units (Axiom Artis, Siemens, Erlangen, Germany). A 6F short sheath with a 6F guiding catheter was used via right-sided femoral access as the standard approach. In cases with severe vessel elongation and the need for an intermediate catheter, an 8F right femoral approach was used. Heparin was administered intravenously immediately after groin puncture (usually 3,000 IU unfractionated heparin IV). All flushing solutions, including the guiding catheters and microcatheters, were heparinized (5,000 IU unfractionated heparin/l).

The p64 was deployed via an Excelsior XT27 (Stryker Neurovascular, Kalamazoo, MI, USA) microcatheter. The diameter and length of the p64 were chosen based on intraprocedural 2D and 3D calibrated measurements of the diameter of the parent artery, the distance between the proximal and distal landing zones, the discrepancy of the diameter between the landing zones, and the aneurysm neck size taking into account potential device foreshortening. Once satisfactory deployment and positioning were achieved, the p64 was mechanically detached.

### Medication

All patients received dual antiplatelet therapy (DAPT) with 1 × 100 mg acetylsalicylic acid (ASA) PO daily and either 1 × 75 mg clopidogrel or 2 × 90 mg ticagrelor or 1 × 10 mg prasugrel PO daily for at least five days before treatment. Alternatively, a loading dose of 500 mg ASA and either 600 mg clopidogrel or 180 mg ticagrelor or 30 mg prasugrel 24h before the treatment was given. From 2012 until 2015 the standard approach was DAPT with ASA and clopidogrel and ticagrelor was only used in case of non-responders to clopidogrel. From 2015 on the standard approach was switched to ASA and ticagrelor to avoid issues due to non-responders to clopidogrel and to achieve a stronger DAPT. Prasugrel was given in case of insufficient DAPT under ticagrelor or in case of intolerance to ticagrelor. The response tests of the antiplatelet regime were done with the Multiplate Analyzer (Roche Diagnostics, Mannheim, Germany) and with the Verify Now (Accriva, San Diego, CA, USA). No p64 was implanted unless adequate platelet inhibition was confirmed. Postprocedural medication consisted of a daily dose of 100 mg ASA and either 1 × 75 mg clopidogrel or 2 × 90 mg ticagrelor or 1 × 10 mg prasugrel PO daily for at least one year. Then DAPT was stopped and switched to 1 × 100 mg ASA PO daily lifelong. According to our institutional protocol, postmedication in patients with an anticipated increased risk of ischemic complications (e.g., if many perforating arteries were covered with an undersized p64) consisted of 2 × 3,000 IU certoparin s.c. (Mono-Embolex, Mylan Healthcare, Germany), daily for 4–6 weeks after flow diversion treatment. The decision to add heparin to the therapy regimen was made based on the device location in perforator-rich areas and the size of the device in relation to the parent vessel.

### Data Collection and Follow-Up

Patency of the parent vessel and distal and side branches and flow characteristics within the flow diverter was assessed angiographically immediately after deployment of the p64 and during follow-ups.

Patients were scheduled for clinical and angiographic follow-up examinations as follows: early follow-up (3–6 months), mid-term follow-up (9–12 months), and long-term follow-up (>24 months). Assessment of the aneurysm occlusion was evaluated according to the O'Kelly-Marotta (OKM) – scale, based on the degree of aneurysm perfusion (7). Adequate occlusion was defined as OKM C and D. Neurological examinations were performed in the peri-procedural period (≤ 24h), post-procedural period (>24 h ≤ 30 days), and during the follow-up (>30 days) by a neurologist or a certified stroke nurse, and recorded using the modified Rankin Scale (mRS) (8). The clinical sequelae of any complication were classified as “transient neurological deficit,” “permanent minor neurological deficit” (i.e., mRS 1 or 2), “permanent major neurological deficit” (i.e., mRS 3–5) or “death.” (9).

## Results

### Patient's Demographics and Aneurysm Characteristics

Between October 2012 and December 2019, a total of 54 patients with 54 aneurysms of the posterior circulation were identified who met the inclusion criteria. There were 45 female patients in our cohort (83 %), and the mean age of the patients was 55.1 years (range 29–76) (Table 1).

**Table 1:** Baseline patient demographics and clinical presentation of the study population.

**Patients characteristics**	
Number of patients	54
Gender (m/f)	9/45
Number of aneurysms	54
**Previously treated aneurysms**	15/54 (27.8%)
Coiling	12
Coiling +Medina	1
WEB	1
Microsurgical clipping (failed)	1
**Aneurysm location**	
Basilar artery bifurcation	10 (18.5%)
Basilar artery trunk	6 (11.1%)
Posterior cerebral artery	3 (5.6%)
Posterior inferior cerebellar artery	18 (33.3%)
Superior cerebellar artery	14 (25.9%)
Vertebral artery (V4)	3 (5.6%)

The mean aneurysm dome size was 3.6 mm (range 0.8–18 mm). Of the 54 aneurysms treated, 51 (94.6 %) aneurysms were small (<10mm), 3 (5.5%) were large (10–25mm), and 0 (0 %) was giant (>25mm). In 51 cases (94.4%), one p64 was implanted, in 2 cases (3.7%), 2 p64 were implanted, and in one case (1.9%), the deployment of a p64 was not feasible even though two different p64 were tried. The implantation of the p64 was the first procedure in 39 aneurysms (72.2%), whereas the remaining 15 aneurysms (27.8%) had been treated before as follows: coiling *n* = 12, coils + Medina *n* = 1, WEB *n* = 1, microsurgical clipping (*n* = 1, the attempted clipping failed). In 10 procedures (18.5%), coils were used as additional devices; in one case (1.9%), additional coiling was attempted but failed. Out of the 54 treated patients 17 patients (31.5%) with an anticipated increased risk of ischemic complications were given additional heparin for 4–6 weeks. Three patients still presented symptoms due to perforator ischemia.

The distribution of the location of the aneurysms in the posterior circulation was: basilar artery (BA)-bifurcation *n* = 10 (18.5%), BA-trunk *n* = 6 (11.1%), posterior cerebral artery (PCA) *n* = 3 (5.6%), posterior inferior cerebellar artery (PICA) *n* = 18 (33.3%), superior cerebellar artery (SCA) *n* = 14 (25.9%) and vertebral artery (VA) intradural *n* = 3 (5.6%) (Table 1).

The mean diameter of the distal landing zone was 2.4 mm (range 1.3–4.4mm), and of the proximal landing zone, 3.0 mm (range 1.6–4.2 mm); the mean discrepancy of the diameter between the proximal and distal landing zone was 0.5 mm, ranging from −1.2 to 1.8 mm.

### Angiographic Follow-Up

An early follow-up (FU) DSA examination (3–6 months) was available for 50 of the 53 (94.3%) aneurysms in which the implantation of the p64 was successful. The FU was performed after a median of 96 days ranging from 42–379 days. The angiographic findings were as follows: complete aneurysm occlusion (OKM D) was observed in 22 (44 %), and a neck remnant (OKM C) in 6 (12%) aneurysms. For 17 (34%) aneurysms, subtotal aneurysmal filling (OKM B) was found. Five aneurysms (10%) remained unchanged (OKM A). In-stent stenosis was found in 3 patients (6%) thereof two moderate ones (50–75%), and one (2%) was graded severe (>75%). All of the patients mentioned above were asymptomatic. Furthermore, there were 2 (4%) side vessel occlusions observed, both asymptomatic.

A mid-term follow-up (9–12 months) was available in 49/53 aneurysms (92.5 %) after a median of 275 days (range 93–522), showing complete aneurysm occlusion (OKM D) in 31/49 (63.3%), neck remnant (OKM C) in 6 (12.3%) aneurysms, and a sac remnant (OKM B) in 8 (16.3 %) aneurysms. Four (8.2 %) aneurysms remained still unchanged (OKM A). In-stent stenosis improved in all cases: 2 (4.1%) were now graded as mild (<50%), and one (2%) was moderate (50–75%). No additional patient developed in-stent stenosis between the first and second FU. There was one other asymptomatic side vessel occlusion (2.0%) observed.

The long-term follow-up (>24-months) was performed after a median of 686 days (range 240–1505) in 41/53 aneurysms (77.4%), revealing complete occlusion (OKM A) in 33 (80.5%), neck remnant (OKM C) in 1 (2.4%), and an aneurysm remnant (OKM B) in the remaining 6 (14.6%), respectively. One aneurysm remained unchanged (2.4%). Therefore, an adequate occlusion (OKM D & C) was seen in 82.9% of aneurysms during the follow-up period. Intimal hyperplasia had resolved entirely, and one new side vessel occlusion (2.4%) had occurred at this FU (Tables 2, 3).

**Table 2:** Angiographic results and occlusion rates.

**Available FU**	
1^st^ FU	50/53 (94.3%)
2^nd^ FU	49/53 (92.5%)
3^rd^ FU	41/53 (77.4%)
**Occlusion rates OKM C+D**	
1^st^ FU	28/50 (56%)
2^nd^ FU	37/49 (75.6%)
3^rd^ FU	34/41 (82.5%)
Parent vessel patency at FU	100 %
Side vessel occlusion at FU	4/53 (7.5%)

*FU, Follow-up; OKM, O'Kelly Marotta. OKM C+D stand for “entry remnant” and “no filling”, and signify sufficient occlusion*.

**Table 3:** Aneurysmal location and angiographic occlusion rates at each time point.

**Location**	**1st FU OKM C+D**	**2nd FU OKM C+D**	**3rd FU OKM C+D**
BA- bifurcation	71.4% (7/50)	85.7% (6/7)	100% (5/5)
BA-trunk	83.3% (5/6)	100% (6/6)	100% (6/6)
PCA	66.7% (2/3)	100% (3/3)	100% (2/2)
PICA	44.4% (8/18)	62.5% (10/17)	78.6% (12/15)
SCA	38.5% (5/13)	61.5% (8/13)	63.9% (7/13)
V4	100% (3/3)	100% (3/3)	100% (2/2)

Retreatment of the target lesion was performed in 5/53 patients (9.4%). In all these patients, the effect of the first implant was considered insufficient, and retreatment was performed by deploying another FDS. Re-treatment was performed if the aneurysms were graded OKM A or B more than 6 months after stopping of DAPT. Of the 5 re-treated aneurysms were three PICA aneurysms and two SCA aneurysms. The jailed vessel had remained patent after the first FD presumably leading to reduced flow diversion effect hence failing to occlude the aneurysm.

**Figure 1:**
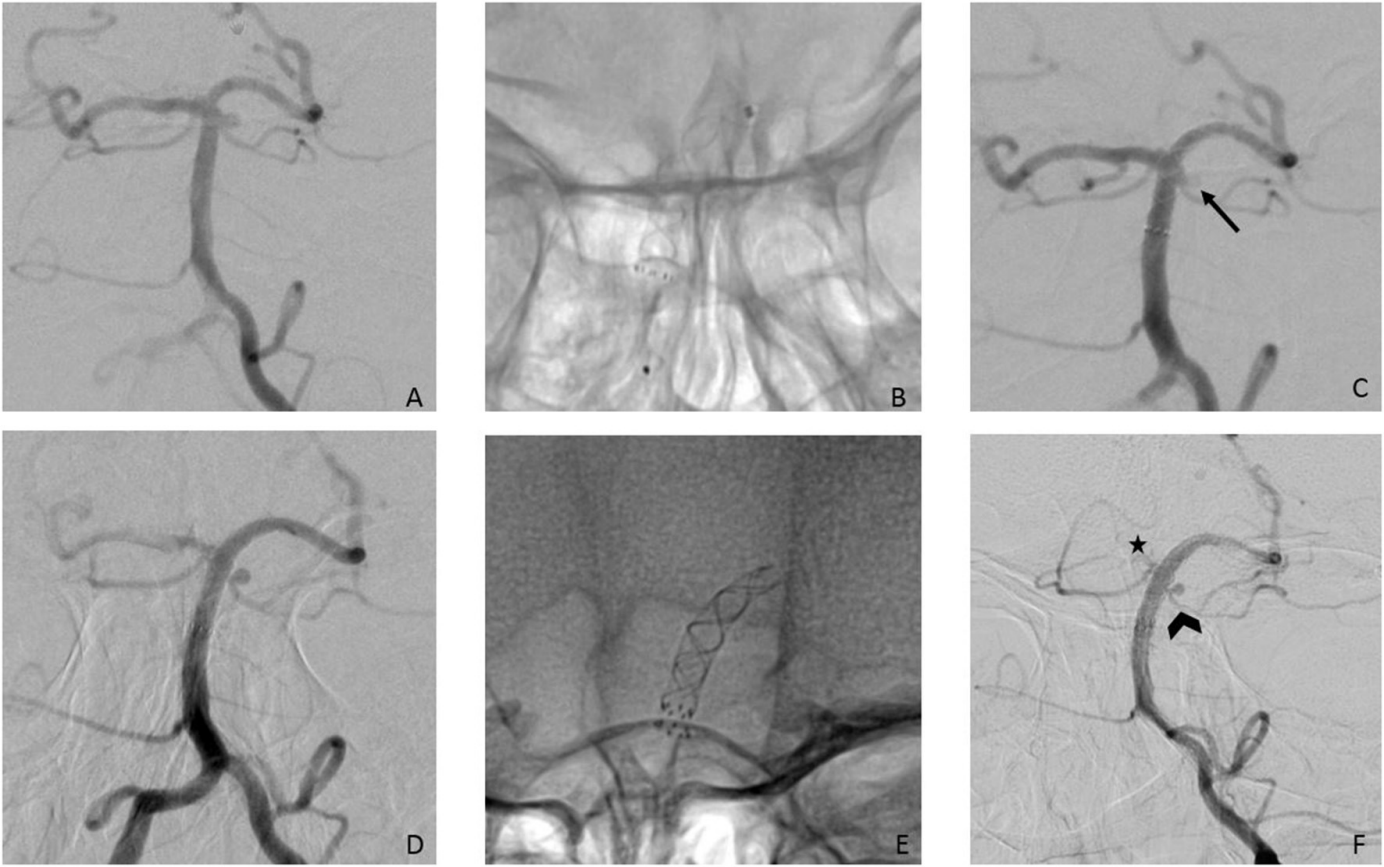
**(A)** Posterior-anterior view after injection of the left VA showing a 2 mm aneurysm of the left SCA; **(B)** successful implantation of a p64 3/12 mm from the left PCA to the BA; **(C)** final run after the FD implantation showed already reduction of the perfusion (arrow); **(D)** 3^rd^ FU after 20 months shows aneurysm unchanged as well as a significant caliber reduction of the right P1- segment. **(E)** a second FD- p64 3/9 mm- was implanted coaxial with the first FD without any complications. **(F)** FU after 12 months after the second confirms that the aneurysm has shrunken, but remains still patent. The left SCA shows significant reduction in size (arrowhead) but is also still patent. The right P1 segment (asterisk) is reduced in caliber now.

During these retreatments, no adverse event occurred. Follow-up DSA examinations confirmed an adequate occlusion (OKM D & C) of the concerning aneurysms in 4 patients. One aneurysm showed reduction in size in follow-up DSA, but was still graded OKM B after the 3^rd^ FU DSA. Patient refused a second re-treatment (Figure 1).

**Figure 2:**
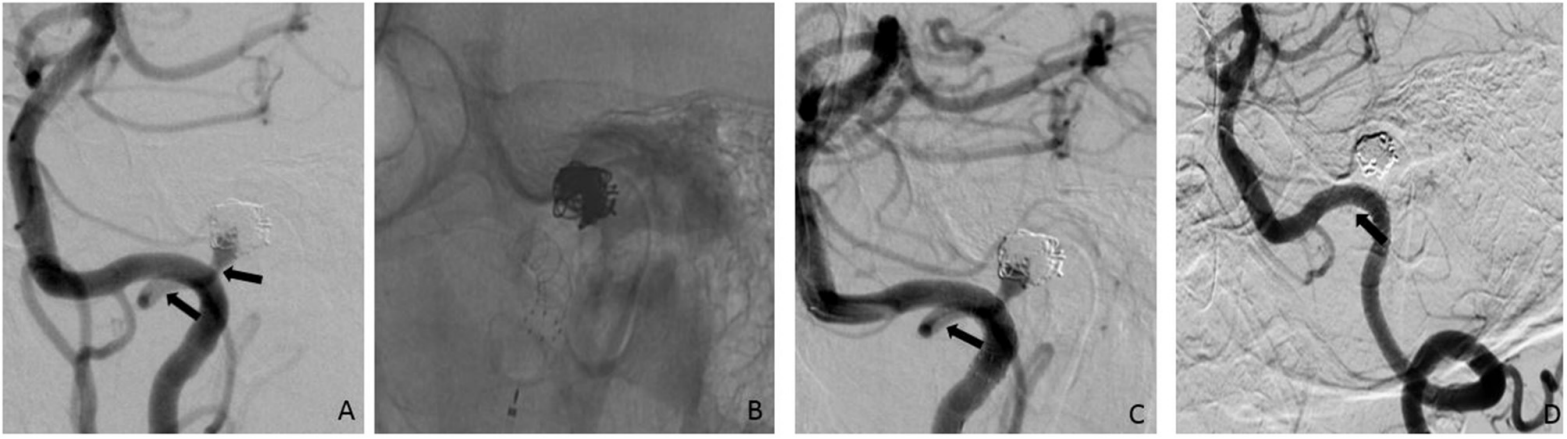
45° LAO injection of the left VA showed a recurrence of a formerly ruptured and coiled left PICA aneurysm; arrows indicating the PICA; **(B)** implantation of a p64 3,5/15 mm; **(C)** final run after the p64 implantation confirmed that the PICA was patent and the aneurysm neck completely covered (arrows = PICA); **(D)** FU angiography after three months reveal an asymptomatic occlusion of the left PICA (arrow) as well as complete occlusion of the aneurysm. LAO, left anterior oblique; VA, vertebral artery; PICA, posterior inferior cerebellar artery.

**Figure 3:**
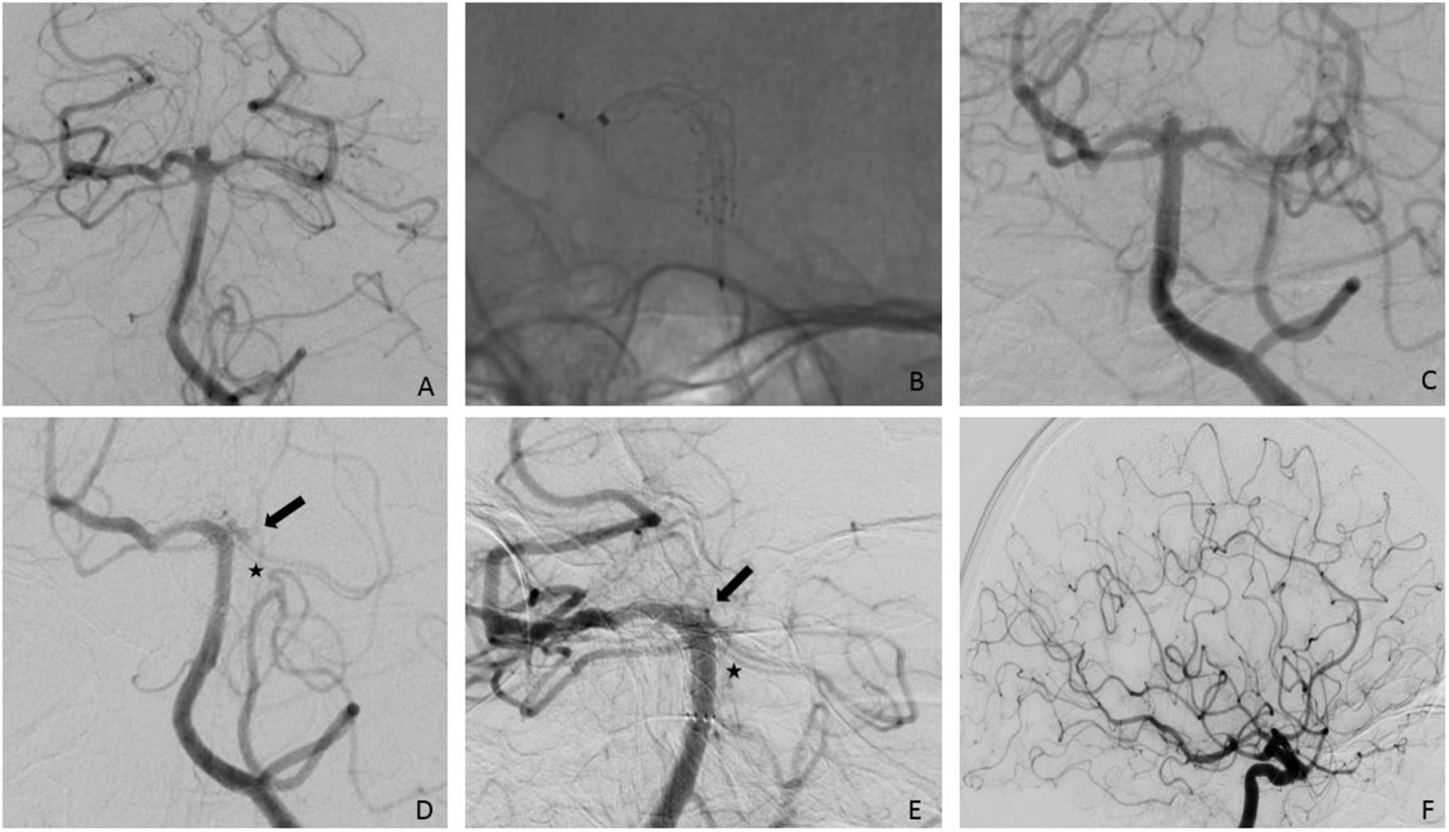
**(A)** Posterior-anterior view after injection of the left VA showing a 2.5 mm aneurysm of the basilar bifurcation, slightly more to the right side; **(B)** successful implantation of a p64 3/15 mm from the right PCA to the BA; **(C)** final run after the FD implantation.; **(D)** first FU after four months shows a small aneurysm remnant as well as a significant caliber reduction of the left P1- segment (arrow). The SCAs on the left side remain the same (asterisk); **(E)**. second FU after nine months shows complete occlusion of the aneurysm as well as the left P1-segment; **(F)**. injection of the left ICA confirms supply of the left PCA via PcomA. pa, posterior-anterior; VA, vertebral artery; PCA, posterior cerebral artery; BA, basilar artery; FD, flow diverter; SCA, superior cerebellar artery; ICA, internal carotid artery; PcomA, posterior communicating artery.

Out of the 53 patients in which the FD implantation had been feasible, angiographically visible side vessel branches had to be covered in 48 patients (90.6%). Acute occlusion of the covered branches was not observed in any of these patients. During the first FU in two patients, the covered branches were occluded (4%), and during the second and third FU, one more side vessel occlusion was observed (2 and 2.4%) (Figure 2). The occluded side vessels were 1 × PICA, 1 × SCA, and 2 × the V4 segment of the VA distal of the PICA. All of these side vessel occlusions were asymptomatic. In 22 patients (41.5%), the origin of the PCA was covered by the p64. In 13 of the 22 cases (59%), the PCA was predominantly or solely supplied by the posterior communicating artery (PcomA) in the FU angiography combined with caliber reduction or occlusion of the P1 segment of the respective PCA (Figure 3). None of these hemodynamic changes were symptomatic.

### Complications

Intraprocedural complications occurred in 2 out of the 54 procedures (3.7%). In one patient, the FD dislocated after detachment, causing incomplete coverage of the aneurysmal neck. Therefore, a second FD was placed.

One patient suffered a subarachnoid hemorrhage (SAH) Hunt & Hess II, Fisher 2 due to a suspected microwire perforation and a dissection of the vertebral artery in the V2 segment that was reconstructed with the implantation of 2 Solitaire Stents (Solitaire AB, Medtronic). The implantation of the p64 failed in this patient due to unstable access. Therefore, the aneurysm was treated with partial coiling of the dome, accepting a neck remnant. The clinical outcome of these patients was rated as mRS 3, equivalent to the condition before the treatment.

Postprocedural complications (24h−30d) were observed in 5 patients (9.3%).

A hemorrhagic complication was observed in one patient (1.9%). This patient suffered a fatal hemorrhage on the day of discharge. The other four patients (7.4%) suffered from symptomatic ischemia resulting in permanent deficits in three patients, and transient symptoms in one patient. A delayed complication (>30 days) occurred in one patient (1.9%) who presented with an internuclear ophthalmoplegia (INO) 15 months after the implantation of the p64 (Tables 4, 5).

**Table 4:** Complications.

**Complications**	
Technical	2/54 (3.7%)
Hemorrhagic	2/54 (3.7%)
Ischemic	5/54 (9.3%)
Transient	1/54 (1.9%)
Permanent	4/54 (7.4%)
Permanent worsening of the mRS	5/54 (9.3%)
Fatal hemorrhage, mrs 6	1/54 (1.9%)
Shift from mrs 0 = > mRS 1	3/54 (5.6%)
Shift from mrs 0 = > mRS 2	1/54 (1.9%)

*mRS, modified Ranking Scale*.

**Table 5:** Clinically relevant complications overview.

**No**.	**Location of the aneurysm**	**Aneurysm size (width × height in mm)**	**device**	**Location of the device**	**Antiplatet regimen**	**Platelet- Inhibition at the time of FD- implantation confirmed with Multiplate or Verify Now**	**Description**	**Transient vs. permanent**	**mRS pre**	**Last mRS**
1	BA tip	4.2 × 3.4	1 × p64 4/21 1 × p64 4.5/21 (both failed)	-	100 mg ASA p.o. 2 × 90 mg ticagrelor p.o.	ADP 12 ASPI 1	postprocedural SAH on CCT, transient drowsiness and headache, resolved spontaneously	Transient	3	3
2	BA tip	2.9 × 2.2	1 × p64 3/15	left PCA to BA	100 mg ASA p.o. 75 mg Plavix p.o.	ADP 47 ASPI 42	Fatal cerebellar IPH on the day of discharge	Permanent	1	6
3	BA trunk	4.6 × 2.3	1 × p64 4.5/15	BA trunk	100 mg ASA p.o. 10 mg prasugrel p.o.	ADP 19 ASPI 8	Bi-lateral pontine perforator infarction with left-sided hemiparesis, dysphagia and dysarthria, at last FU remaining mild dysarthria	Permanent	0	1
4	SCA	1.8 × 1.0	1 × p64 3/15	left PCA to BA	100 mg ASA p.o., 75 mg Plavix p.o.	ADP 7 ASPI 6	INO 15 months after treatment, 3 month after stop of DAPT	Permanent	0	1
5	PICA	2.3 × 2.1	1 × p64 3.5/15	right V4 segment	100 mg ASA p.o. 75 mg Plavix p.o.	ADP 71 ASPI 19 ARU 391 P2Y12 (20/139) = 92 %	Scattered diffusion restriction in both cerebellar hemispheres with vertigo, vomiting and dizziness	Transient	0	0
6	SCA	1.8 × 2.2	1 × p64 3.5/15	left PCA to BA	100 mg ASA p.o. 75 mg Plavix p.o.	ADP 25 ASPI 6	Bilateral pontine perforator infarction with right-sided hemiparesis	Permanent	0	2
7	BA tip	1.5 × 1.2	1 × p64 3/12	right PCA to BA	100 mg ASA p.o. 2 × 90 mg ticagrelor p.o.	ADP 13 ASPI 6	Left pontine perforator infarction with discrete finger movement impairment	Permanent	0	1

*BA, basilar artery; SAH, subarachnoid hemorrhage; CCT, cranial computed tomography; mRS, modified Ranking Scale; IPH, intra-parenchymal hemorrhage; INO, internuclear ophthalmoplegia; SCA, superior cerebellar artery; PICA, posterior inferior cerebellar artery; ASA, acetylisalicylic acid; p.o., per os*.

We did not observe any device-related thromboembolic complications in our series during the procedural and postprocedural period nor any in-stent thrombosis.

### Clinical Follow-Up

Clinical deterioration with a permanent worsening of the mRS was reported in 5 of the 54 patients (9.3 %) as follows: one fatal hemorrhage (mRS 6, 1.9%), one shift of the mRS from 0 =>2 (1.9%), and 3 patients with a shift from mRS 0 =>1 (5.6 %) (Table 3).

Tables 2, 3 show a breakdown of aneurysm occlusion rates and Tables 4, 5 of complication rate.

## Discussion

Flow diversion has gained an essential role in treating IAs in the last decade, especially in wide-necked, giant, or fusiform aneurysms. This is even more distinct for the so-called “challenging” aneurysm in the anterior circulation. The implantation of FDs for the treatment of posterior circulation aneurysms remains controversial due to the significantly higher rates of ischemic complications and higher morbidity and mortality that have been reported throughout the literature (4, 6, 10). However, the composition of the respective study populations has to be considered as they did not solely focus on saccular aneurysms but also included giant, fusiform, and dissecting aneurysms. It has been shown that these non-saccular aneurysms are especially prone to ischemic complications due to extensive thrombus formation (5, 11). In this series, only saccular aneurysms were included. We observed ischemic complications in 4/54 patients (7.4%), with 1/54 (1.9%) patients suffering a deterioration of the mRS from 0=>2. According to previously published data, these results confirm that smaller (usually saccular) aneurysms carry a lower risk of ischemic complications (12).

The porosity of the FD is another crucial factor regarding ischemic complications and aneurysm occlusion rates. The porosity is defined as the ratio of metal-free surface area to the entire surface area, and pore density is the number of pores per unit surface. It has been shown that FD with a porosity of 70% achieves a high rate of aneurysm occlusion without affecting the covered branches (13).

The p64 is braided using 64 nitinol wires with a lower porosity of 51–60%, depending on the device size in relation to the parent vessel diameter (14). Lower porosity is supposed to lead to higher occlusion rates but might also be prone to more ischemic complications. Aguilar et al. reported high complete or near-complete occlusion rates of 89.7% at mid-term FU and 94.5 % at long-term FU (>24 months) in saccular aneurysms in the anterior circulation in the most extensive study with the p64 from a single center (2). Despite the higher porosity of the p64, they observed similar ischemic complication rates- reported as 4.8% in their study- that have been reported for other FDs such as the Pipeline Embolization Device (PED) (Medtronic, Dublin, Ireland), Silk (Balt Extrusion, Montmorency, France), and Surpass (Stryker Neurovascular, Fremont, CA, USA) (15). As compared to these data, we observed a higher rate of ischemic complication of 9.3 % overall in this study, as is to be expected due to the increased vulnerability of the covered perforating arteries of the posterior circulation compared to FD in the anterior circulation. However, there was only one major stroke (1.9%) despite the higher porosity of the p64. In comparison, Bender et al. report major stroke complications of 8% with the PED in the posterior circulation, and Kulcsar et al. report 25% with the SILK FD (12, 16). Despite the higher porosity of p64, the significantly lower rate of severe stroke complications might be attributed to the device's sizing. We always choose to slightly oversize the FD, avoiding foreshortening and preserving perforating arteries. In addition, for patients with a high risk of perforator stroke, low molecular weight heparin is administered for 4–6 weeks after the FD implantation in addition to the DAPT.

In our analysis concerning occlusion rate, we observed a progressive increase in aneurysm occlusion with a complete or near-complete obliteration rate of 56% at 3-month, 75.6% at 9-month, and 82.5% at the last angiographic follow-up. The lowest occlusion rates at the last FU were observed for PICA aneurysms with 78.6 % and SCA aneurysms with 63.3%. These low occlusion rates might be because the parent vessels stayed patent in most cases, leading to a reduced flow-diverting effect by sucking blood from the BA or VA to the respective parent vessel, hence failing to occlude the aneurysm. Similar observations have been reported for fetal PcomA aneurysms (2, 17). Occlusion rates, regardless of location, have been reported very heterogeneously in literature ranging from approximately 50% up to 96–100 % even (5, 16, 18). The meta-analysis of Brinjikji et al. analyzed 29 articles with 1,451 patients and 1,654 aneurysms and found an occlusion rate of 80% (69–88 %) for small posterior circulation aneurysms (11). The wide range of reported occlusion rates reflects the heterogeneous nature of posterior circulation aneurysms as almost all the studies also included giant and fusiform aneurysms. Another reason might be the small study populations in some of the articles prone to statistically less meaningful data. Our results are in line with the meta-analysis as mentioned above. Oversizing of the device did not lead to a significantly lower occlusion rate while preserving the perforating arteries. Therefore moderate oversizing of the FD seems to be an essential factor for the treatment in posterior circulation aneurysms, achieving a reasonable occlusion rate without increasing the risk of complications. As concerning complications, it has been suggested that the lower porosity of p64 may lead to a higher rate of ischemic complications, which is primarily an essential concern in the posterior circulation. However, an increased number of ischemic complications was not observed in our series, with a total of 5 events (9.3%) consisting of 4 permanent minor neurological deficits (7.4%) and one case (1.9%) of transient neurological symptoms. We observed no permanent major neurological deficit. There was one device-unrelated, treatment-related mortality of 1.9%. This is significantly lower than previous studies reported for FD in the posterior circulation (4, 10). Bender et al. report major stroke complications in 8% (12). Toth et al. report mortality rates and permanent neurological deficits of each 14.3 % with the PED in predominantly saccular aneurysms (19). These numbers are significantly higher than the results from our series with the p64 that carries a supposedly higher risk of ischemic complications. Possible explanations may be the oversizing of the device and the additional use of low molecular weight heparin combined with DAPT during the first 4 to 6 weeks after FD implantation as bridging of the most thrombogenic and, therefore, most vulnerable period after FD implantation.

Of course, DAPT in combination with heparin poses an increased risk for hemorrhagic complications. Hemorrhagic transformation of clinically silent microinfarcts observed after endovascular procedures have been reported as one of the significant drawbacks of FD implantation (20–22). Our series report one fatal hemorrhage three days after the p64 implantation, most likely to be attributed to delayed parenchymal hemorrhage due to microischemia. Additionally, this patient had been a hyper-responder for clopidogrel.

The recent introduction of surface- modified, hydrophilic coated FDs such as the p64MW HPC and p48MW HPC (phenox, Bochum, Germany) and the Pipeline Shield (Medtronic, Dublin, Ireland) allows the implantation of FD under single platelet inhibition (SAPT) only (23–25).

## Limitations

The limitations of this study include those inherent in a retrospective study, and this study reports the experience of a single center with a single specific flow diverter. Secondly, this study dealt with unruptured, saccular aneurysms of the posterior circulation only, explicitly excluding fusiform, giant, and dissecting aneurysms of the posterior circulation.

## Conclusion

The p64 is safe and effective for treating intracranial saccular unruptured aneurysms arising from the posterior circulation with acceptable occlusion rates on mid-term and long-term follow-ups and low morbi-mortality rate. This represents the most extensive study to date on the use of p64 in saccular posterior circulation aneurysms exclusively.

## Data Availability Statement

The raw data supporting the conclusions of this article will be made available by the authors, without undue reservation.

## Ethics Statement

The studies involving human participants were reviewed and approved by Ethikkommission der Landesärztekammer Baden-Württemberg. The patients/participants provided their written informed consent to participate in this study. Written informed consent was obtained from the individual(s) for the publication of any potentially identifiable images or data included in this article.

## Author Contributions

MA-P, EH, CS-C, and VH: data gathering and manuscript preparation. CW, OG, and HB: review and editing. HH: guarantor, overall review, and study design. All authors contributed to the article and approved the submitted version.

## Conflict of Interest

MA-P and VH serve as proctors and consultants for phenox. HH is a co-founder and share-holder of phenox and femtos. The remaining authors declare that the research was conducted in the absence of any commercial or financial relationships that could be construed as a potential conflict of interest.
